# Anthocyanins, Carotenoids and Chlorophylls in Edible Plant Leaves Unveiled by Tandem Mass Spectrometry

**DOI:** 10.3390/foods11131924

**Published:** 2022-06-28

**Authors:** Clara Sousa

**Affiliations:** CBQF—Centro de Biotecnologia e Química Fina—Laboratório Associado, Escola Superior de Biotecnologia, Universidade Católica Portuguesa, Rua Diogo Botelho 1327, 4169-005 Porto, Portugal; cssousa@ucp.pt

**Keywords:** plant leaves, food, pigments, metabolomics, mass spectrometry, chemometrics, fingerprint

## Abstract

Natural pigments are a quite relevant group of molecules that are widely distributed in nature, possessing a significant role in our daily lives. Besides their colors, natural pigments are currently recognized as having relevant biological properties associated with health benefits, such as anti-tumor, anti-atherogenicity, anti-aging and anti-inflammatory activities, among others. Some of these compounds are easily associated with specific fruits (such as blueberries with anthocyanins, red pitaya with betalain or tomato with lycopene), vegetables (carrots with carotenoids), plant leaves (chlorophylls in green leaves or carotenoids in yellow and red autumn leaves) and even the muscle tissue of vertebrates (such as myoglobin). Despite being less popular as natural pigment sources, edible plant leaves possess a high variety of chlorophylls, as well as a high variety of carotenoids and anthocyanins. The purpose of this review is to critically analyze the whole workflow employed to identify and quantify the most common natural pigments (anthocyanin, carotenoids and chlorophylls) in edible plant leaves using tandem mass spectrometry. Across the literature there, is a lack of consistency in the methods used to extract and analyze these compounds, and this review aims to surpass this issue. Additionally, mass spectrometry has stood out in the context of metabolomics, currently being a widely employed technique in this field. For the three pigments classes, the following steps will be scrutinized: (i) sample pre-preparation, including the solvents and extraction conditions; (ii) details of the chromatographic separation and mass spectrometry experiments (iii) pigment identification and quantification.

## 1. Introduction

Pigments are certainly among the most impactful compounds in our lives. They can be classified as being of natural (produced by living organisms) [1,2,3,4] or synthetic origin (obtained in laboratory) [5,6]. Natural pigments bring color to our lives through their presence in leaves, fruits, vegetables, flowers, human skin, blood, eyes, bacteria and fungi. However, beyond beauty, they possess crucial functions in the life cycle of several organisms. In plants, chlorophyll and carotenoids enable photosynthesis [7], quinones convert light in energy [8] and flavonoids are produced under stress conditions [9]. In the animalia kingdom, myoglobin and hemoglobin allow life through oxygen transport [10] and melanin acts as a screen protector [11]. Natural pigments also seem to be associated with several health benefits, such as anti-tumor, anti-atherogenicity, anti-aging and anti-inflammatory activities [12,13,14]. Due to their relevance, natural pigments have been the target of many studies involving: (i) their identification or quantification; (ii) unveiling how they differ across species or plant cultivars and microorganisms strains; (iii) the clarification of how they are affected by the plants’ growing conditions; (iv) the elucidation of their biological properties (anti-tumoral, anti-microbial and anti-oxidant, among others); and (v) the clarification of their biosynthetic pathways or how genetic mutations impact their biosynthesis [15,16,17,18,19,20,21].

Since early civilization, pigments have been used for many purposes, giving color to manufactured products and being used to process food, medicines (even without any sense of food safety) and cosmetics. Nowadays, these compounds are still used as food colorants to preserve or intensify the natural color of food, which is associated with quality, and to confer an attractive appearance or to restore color loss during storage in the cosmetic industry or in dyes for textiles or paints [4,22,23,24,25,26,27]. Pigments are molecules able to absorb light in the visible region. Such molecules possess a chromophore (part of the pigment molecule), which can selectively capture specific wavelengths. The remaining wavelengths of the visible spectrum are reflected or refracted and perceived by the human eye as color. The chromophores could be double-bond conjugated systems such as those present in anthocyanins, carotenoids and betalains or metal-coordinated porphyrins such as chlorophylls and myoglobin.

Anthocyanins are phenolic water-soluble glycosides or acyl-glycosides of anthocyanidins [28]. These compounds are secondary plant metabolites, protecting them against biotic and abiotic stresses, being the most abundant cyanidin delphinidin and pelargonidin derivatives. Anthocyanins are responsible for the pink, red, blue and purple colors in flowers, fruits and vegetables, with the color being related to the substitution pattern (position and chemical groups) on the aromatic rings. They have been used as natural food coloring agents and are emerging as promising ingredients in food and nutraceutical industries [29]. Anthocyanins are also recognized as possessing anti-microbial, anti-cancer and anti-diabetic activities [30,31,32] and as potent antioxidant and anti-inflammatory agents [33,34].

Carotenoids are another set of naturally occurring plant pigments, mostly responsible for the red, yellow and orange colors of vegetables and autumn leaves (when all the chlorophyll has already been degraded), but can also be found in dark green vegetables [35]. This class of pigments possess a common C40-backbone with a system of conjugated double bonds, which are classified as carotenes (only containing carbon and hydrogen) or as xanthophylls (containing additionally oxygen) [36]. Carotenoids are synthetized mostly by photosynthetic organisms such as plants and algae, as well as some microorganisms such as fungi and bacteria. They possess several relevant functions in the Plantae kingdom, acting as light harvesters, regulators of growth, inhibitors of photooxidation during photosynthesis and attractors of pollination agents due to their vivid colors [37,38]. The most common types in plant leaves are lutein, β-carotene, violaxanthin and neoxantin. The consumption of these compounds by humans, who are not able to synthetize them, seems to have a prominent role in reductions in several diseases such as eye-related cancer, immune disorders and cerebrovascular and cardiovascular diseases [39]. Carotenoids are also quite relevant in industry, being used as food colorants, cosmetic products and nutraceuticals [40].

Another relevant pigments class contains chlorophylls, which are present in photosynthetic organisms such as plants, algae and cyanobacteria [41]. These pigments are large molecules with a cyclic part (chlorine ring) bound to a metal ion (magnesium), which reflects green light. Five forms of chlorophyll are known (chlorophylls a, b, c, d and f), presenting slightly distinct functions during the photosynthetic processes undertaken by the different organisms. In plants, during senescence and fruit ripening, the programed chlorophyll breakdown occurs to allow the remobilization of nutrients to parts of the plant that are still growing [42]. This phenomenon unmasks the presence of carotenoids and anthocyanins in green plant leaves, which are mainly observed in autumn.

Additionally, mass spectrometry (MS) has gathering more attention in the last years due to the emerging OMICS concept, and is currently a widely used technique for many purposes, such as for plant metabolome characterization, including for pigments.

**Figure 1:**
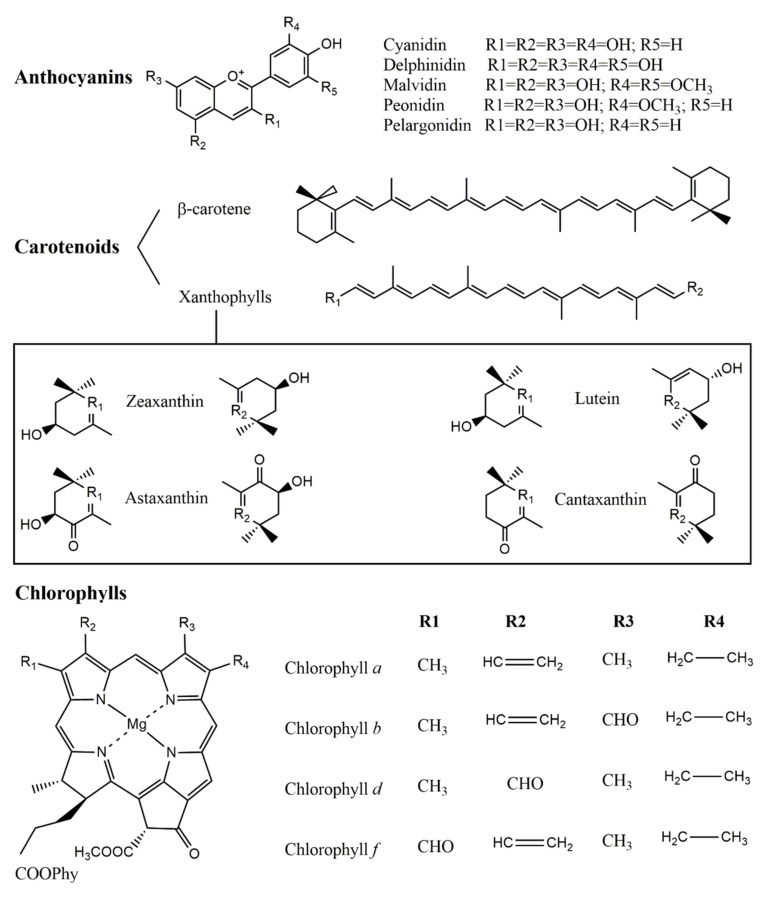
Chemical structures of the most commonly found pigments in plant leaves.

This review will be focused on the identification of anthocyanins, carotenoids and chlorophylls (Figure 1) in edible plant leaves through tandem mass spectrometry, and includes: (i) an introductory part presenting the main characteristics of natural pigments, focused on anthocyanins, carotenoids and chlorophylls and the fundamentals of mass spectrometry, as well as the corresponding data analysis; (ii) a review section; (iii) a conclusion and critical analysis of the published studies. In the review section, the three focused pigment classes are described separately, including a discussion about the sample pre-processing and extraction conditions (solvents used, sample/solvent ratios and extraction procedures), the mass spectrometry technique and ionization type, the prior chromatographic separation process when used (type of chromatographic column and eluents) and the identified pigments, as well as the data analysis tools and databases used.

## 2. Natural Pigments

### Mass Spectrometry: Main Principles, Advantages and Disadvantages

Mass spectrometry (MS) is a quite sensitive technique that measures the mass-to-charge (*m/z)* ratio of ions, aiming for their identification or quantification in simple or complex samples. The basic components of a mass spectrometer are a sample introduction gateway, an ionization source, a mass analyzer and a detector, plus a data acquisition system. Briefly, mass spectrometry experiments include the introduction of a sample into a vacuum system, which is subsequently pushed throughout all spectrometer components until reaching the detector [43]. Samples can be introduced into the system via direct infusion or from a previous chromatographic separation step.

Once inside the vacuum system, sample ionization is mandatory, as neutral species cannot be steered by the electric fields employed in mass spectrometers. There are many ion sources that are used according to the type of sample, target analyte and application. The most common ionization methods are [43]: (i) gas-phase methods (electron ionization—EI; chemical ionization—CI; direct analysis in real time—DART; inductively coupled plasma—ICP), (ii) desorption methods (matrix-assisted laser desorption ionization—MALDI; fast-atom bombardment—FAB; thermal ionization sources and plasma ionization sources; liquid metal ion sources—LMIS) and (iii) spray methods (electrospray ionization—ESI; desorption electrospray ionization—DESI). Among the gas-phase methods, EI is a fairly harsh ionization method mostly that is applied to volatile and low-molecular weight molecules. CI is a very soft ionization method, based on the interaction of an analyte and a carrier gas, leading to a reduced fragmentation pattern, which is easier to interpret but also reduces the analyte’s structural information. In the DART method, nitrogen or helium plasma firstly produces excited-state species, which interact with the analyte, promoting its ionization. This is a very fast ionization method, being suitable for rapid analysis. This method is commonly used in forensics and food analysis, and the main advantages are its speed, high salt tolerance and low or no sample preparation requirements [44]. The ICP method is typically applied to liquid samples that have been previously converted into aerosols, which are further introduced in an argon gas plasma. Regarding the desorption ionization methods, MALDI is among the most used methods, being considered one of the major soft ionization methods. It is of particular relevance for large biomolecules such as proteins, peptides, polymers and lipids, and demands the use of a matrix chosen according to the type of analyte. The sample plus the matrix is irradiated by a laser leading to the ionization molecule. FAB is also a soft ionization method based on the interaction of an accelerated atom beam with an analyte. It also uses a matrix, commonly glycerol, to protect samples, to prevent aggregation, and as in MALDI, to promote ionization. Thermal and plasma ionization sources and LMIS are rarely used and will not be addressed here. The spray methods are among the most used ionization methods. ESI is a soft method suitable for large molecules, in which the analyte in the solution is conducted through a high-voltage capillary producing charged droplets. The DESI method is quite similar to the ESI but the charged droplets are formed previously and further directed to a sample, promoting its desorption [45]. This ionization method is characterized by its high speed, low fragmentation occurrence, molecular specificity, high sensitivity, low matrix and salt sensitivity, virtual applicability to all compounds classes and quantitative accuracy and precision.

After the ionization step, the ionized analytes are pushed to the mass analyzer, allowing their separation according to their masses. The most commonly used analyzers are [43] time-of-flight (TOF), quadrupole, ion trap and orbitrap analyzers. TOF mass analyzers are based on the time taken by each ion to travel to the flight tube and reach the detector. This time is proportional to the *m/z* ratio once smaller ions travel faster, arriving at the detector first. TOF systems have a very good mass resolution and are able to acquire a very wide range of *m/z* values. In the quadrupole analyzers, a radio frequency is applied between two pairs of metal rods and the ion trajectory is deflected according to their *m/z,* enabling their separation. The advantages of this kind of analyzer include the good scan speed and sensitivity, plus a mass range of up to 2000 *m/z* and high-speed polarity switching. On the other hand, they have quite poor mass resolutions if used as a single system. The ion trap mass analyzers are very similar to the quadrupole ones; however, the separation is based on discharging ions with unstable oscillations from the system to the detector. The main advantages of this kind of mass analyzer are their small size and relative low cost, while still possessing good sensitivity and resolution. The orbitrap analyzers are based on the orbital ion movement of each ion around an inner spindle-like electrode. The main advantages of the orbitrap analyzers are their very high mass resolution and accuracy.

Tandem mass spectrometry (tandem MS) or MS/MS is a particular case of MS that involves two or more mass analyzers separated by a collision cell. In the collision cell, ions are fragmented and the generated fragments can be attributed to specific chemical structures.

After passing through the mass analyzer(s), the ions reach the detector, the main functions of which are ion detection and signal amplification. The most commonly used detectors are [43] electron multiplier (EM), Faraday cup (FC), photomultiplier conversion dynode and array detectors. No deep details will be given about their operation principles because they are not relevant to this review.

## 3. Data Analysis

Countless amounts of data are produced in a short time period with modern analytical instruments for large sample sets, making data analysis a crucial task. Depending on the undertaken experimental approach, data obtained through mass spectrometry present distinct complexity levels and could be analyzed with different tools. Chemometrics is among the most frequently selected approaches, enabling sample differentiation based on the metabolite pattern using unsupervised methods such as a principal component analysis (PCA) or hierarchical clustering analysis (HCA). Supervised approaches [46], such as linear discriminant analysis (LDA), partial least squares discriminate analysis (PLSDA), k-nearest neighbors, soft independent modeling class analogy (SIMCA), support vector machines (SVM) and artificial neural networks (ANN), are also used to unveil metabolic differences between pre-defined samples. Both approaches, i.e., supervised and non-supervised methods, are mostly used for sample clustering based on the different metabolic fingerprints or to select discriminatory mass features among samples. A further biological interpretation requires the identification of specific metabolites for each sample cluster (biomarkers). Biomarker identification is mostly carried out through comparisons between the obtained mass spectra (exact mass and fragments) and the ones present in available databases (which could be from experimental studies or obtained in silico). The most commonly used databases are the KEGG Ligand Database [47], PubChem Project [48], Human Metabolome Database (HMBD) [49], Metabolome Japan [50], METLIN database [51], NIST databse [52] and Mascot [53], among others. Other additional and online available tools such as Metaboanalyst [54] Global Natural Products Social Molecular Networking (GNPS) [55], Mzmine [56] and Cytoscape [57] are very helpful for mass spectra analysis.

## 4. Mass Spectrometry Analysis of Leaves Pigments

### 4.1. Antocyanins

#### 4.1.1. Samples, Preprocessing and Extraction Details

Anthocyanin identification and quantification processes were undertaken in fresh and dried leaves (Table 1). Previous dried leaf studies used commercially available samples [58] or laboratory-dried ones [9,10,11,12,13,14,15,16,17,18,19,20,21,22,23,24,25,26,27,28,29,30,31,32,33,34,35,36,37,38,39,40,41,42,43,44,45,46,47,48,49,50,51,52,53,54,55,56,57,58,59,60,61,62,63,64,65,66,67,68,69,70,71,72]. Gómez-Martínez et al. [69] and Zhang et al. [70] mentioned the use of shade, while the remaining authors did not give any details about the drying process. Among the published studies using fresh leaves, the leaves were immediately lyophilized [61,62,63,64,65,66] or frozen in liquid nitrogen [16,20,59,60,67,68]. The apparent immediacy of leaf processing and analysis clearly points to a possible degradation of this compound class. Regarding the extraction solvents, methanol was by far the most used, almost always in the form of an aqueous solution under acidic conditions. The selection of methanol is somehow expected once anthocyanins are polar in nature, with the extraction being facilitated by similar (polar) solvents. Other chemicals were added in small amounts by some authors for different purposes. Viacava et al. [63] added ascorbic acid to their samples during the extraction process to prevent polyphenol oxidation by the polyphenoloxidase enzyme. A single study used acetone [67] and another used ethanol [69]. Additionally, one study was found to use quite unusual solvents, namely natural deep eutectic solvents [72]. No studies were found comparing different solvent systems for anthocyanin extraction, which could be valuable information. Among the published studies, most of them provided detailed information about the sample/solvent ratios used, which varied from around 20 to 100 mg/mL. Jiang et al. [58] did not mention the volume of the solvent used, while McCance et al. [20] referred to the use of a “known” mass of sample. Regarding the extraction procedure itself, a lot of different procedures were reported by the authors. They all involved an initial step consisting of a period of time in which the samples were placed in contact with the main extraction solvent. However, the period of time, temperature and conditions (simple maceration, ultrasonic bath or others) were very different. After this step, some of the authors simply centrifuged the samples [16,59,60,61,62,69], while others went ahead with more or less complex and laborious re-extraction procedures [20,66,72]. Similarly to the extraction of solvents, no studies were found comparing the different extraction procedures, not even involving slight modifications such as time (sample–solvent contact) or temperature changes.

#### 4.1.2. Mass Spectra Acquisition

Mass spectra acquisition (Table 2) was mostly preceded by a liquid chromatographic separation step [16,20,58,59,60,61,62,63,64,65,66,67,68,69,70,72]. Uarrota et al. [71] used a MALDI-TOF MS system in which samples were loaded in the analysis plate directly. When used and specified, the separation step occurred in reversed-phase (RP) C18 columns with different lengths, diameters and particle sizes, namely (30–250) × (2.1–4.6) mm, with particle sizes in the range of 1.7–5 µm. No comparisons among columns were reported in any of the studies. Regarding the elution system, all of the studies employed an elution gradient combining water with acetonitrile or methanol. The eluents were always acidified with small amounts of formic acid (0.1–1%) [16,20,58,59,61,62,64,65,66,67,68,69,70,72] or acetic acid [60,63]. Similarly to the columns used, none of the studies gave details about the choice or optimization of the elution system.

All of the studies but one were undertaken using the same ionization source, ESI, which was operated mostly in the positive mode. Qin et al. [62], Viacava et al. [63] and Simirgiotis et al. [66] employed both modes, ESI + and ESI −, while Gómez-Martínez et al. [69] solely used the negative mode. This class of compounds should be easily ionized by losing a proton. A single study used MALDI to ionize samples [71]. In all of the studies, the ionization type was mentioned without any additional information as to whether a choice was made or if was the only option available. Regarding the mass analyzers, the most commonly employed were QTOF analyzers [16,20,62,63,70,72], used in 6 studies. The remaining studies used other mass analyzers such as orbitrap [60] or ion trap [61] analyzers, while some authors did not clearly specify.

#### 4.1.3. Identified and Quantified Anthocyanins

Anthocyanin and its derivatives were identified with more or less complex approaches in the published studies (Table 3). Some authors used the chromatographic (mostly the retention time (RT)) and mass (parent ion and mass fragments) parameters alone [63] or combined with other techniques [58] for metabolite identification. Other studies included available online databases [60,61] combined or not with a previous chemometrics step [16,59]. A single study [71] used mass spectra obtained from MALDI-TOF MS (without a previous separation chromatographic method) to perform metabolite identification in *Zea mays* leaves from eight landraces. Seven anthocyanin compounds were identified solely through the typical signal (*m/z*) for each compound. It should be noted that the authors recognize the difficulty of using this technique, which is primarily designed for high-molecular weight compound analyses and for low-molecular weight metabolite identification due to the signal overlapping with the peaks from the matrix.

The published studies involving metabolite identification from chromatographic data (RT) and parent ion plus mass fragment information (without chemometrics or online databases) have focused on several plant species with quite different goals. Viacava et al. [63] performed a deep metabolic study on red and green oak *Lactuca sativa* leaves and identified four cyanidin derivatives in the red oak cultivar, namely cyanidin-3-*o*-glucoside, cyanidin-3-*o*-(6″-*o*-acetyl)-glucoside, cyanidin-3-*o*-(3″-*o*-malonyl)-glucoside and cyanidin-3-*o*-(6″-*o*-malonyl)-glucoside. It should be stressed that the first two compounds were identified only through the chromatographic parameters because they were not detected in the MS experiments. The authors claimed that they performed the ESI in the negative mode, which prevented the detection of low-content anthocyanins, as it is known that these compounds are much better ionized in the positive mode. Jiang et al. [58] developed a quite complex and laborious procedure to isolate four anthocyanin compounds in a commercial tea. The authors did not use MS for deep sample screening but rather to elucidate the structures of the isolated compounds (via parent ions and fragments), together with the results from other techniques (RT, ^1^H and ^13^C NMR analysis). A single sample was included in the study and 2 cyanidin and 2 delphinidin derivatives were identified. Yuan et al. [64] evaluated the anthocyanin content of the buds from a new kind of *Lonicera japonica*, also a popular tea plant, and identified 8 cyanidin derivatives using RT, λmax, parent ion and fragment mass information, with the most abundant being cyanidin-3,5-diglucoside and cyanidin-3-glucoside. Simirgiotis et al. [66] analyzed *Fragraria chiloensis* fruit, leaves and rhizomes extracts and performed a tentative compound identification process. Globally, authors have identified several procyanidin tetramers in leaf extracts with different masses and at different RTs, but were not able to unequivocally identify them. Hijaz et al. [67] have studied transgenic ‘Mexican lime’ leaves and identified 9 anthocyanins compounds (6 cyanidins, 2 delphinidins and 1 peonidin derivatives) in their samples also without the use of databases nor chemometric tools. For some compounds, the authors were not able to totally elucidate their structure. McCance et al. [29] evaluated the effect of plant maturity in the anthocyanins content of three *Ocimum basilicum cultivars* and four highly abundant cyanindin derivatives were identified.

Metabolite identification was performed by 7 authors using RT, parent ion and mass fragments plus available online databases [16,61,65,68,69,70,72]. The databases used included KNApSAck, METLIN, MassBank, Metware, MWDB, HMDB, Varian MS Workstation, MoNA, FoodDB, Lipid Maps and PlantCyc. Zhang et al. [60] compared the alterations in leaf metabolites of *Camellia sinensis* induced by leaf color changes. Two anthocyanin derivatives (proanthocyanidin III; delphinidin–hexose–coumaroyl) were identified and related to the leaf color changes. Shen et al. [61] evaluated the temperature stress effects (ML—moderatelly low; SL—severely low; MH—moderately high; SH-—severely high) in the metabolic profiles of the leaves. The authors identified five anthocyanin monomers (cyanidin-3-*o*-glucoside, cyanidin *o*-hexosyl-*o*-hexosyl-*o*-hexoside, cyanidin *o*-syringic acid, cyanidin-3-*o*-glucoside chloride and cyanidin 3-galactoside) that significantly accumulated SL temperature stress. Goh et al. [65] evaluated the temperature effect on the metabolite profiles of *Polygonum minus* from different lowland and highland origins and concluded that among the eight identified anthocyanins, significantly less compounds were detected under higher temperature treatments. Deng and co-workers [68] studied the juvenile red fading phenomenon of sweet potato leaves (*Ipomea batatas*) and identified five anthocyanins (2 cyanidin and 3 peonidin derivatives), the contents of which significantly varied during the process. Gómez-Martínez et al. [69] evaluated the composition of *Moringa oleifera* leaflet extracts planted in three distinct locations and identified, among other phenolic compounds, two anthocyanin compounds (cyanidin 3,5-*o*-diglucoside and peonidin 3-*o*-(6″-acetyl-glucoside)). It should be stressed that these two compounds were found in the leaflets obtained from the three locations. Zhang et al. [70] performed a deep phenolic profiling of *Cydonia oblonga* leaves and putatively identified 47 anthocyanins among a total of 275 phenolic compounds using an untargeted metabolomics approach. However, from these 46, the authors were only able to confirm via tandem MS the structures of three (cyanidin, cyanidin-3-glucoside and delphinidin 3-galactoside). Bentley and colleagues [61] explored the differences in the anthocyanins profiles of *Myrothamnus flabellifolia* samples obtained using natural deep eutectic solvents and were able to structurally elucidate 9 anthocyanin compounds, which differed in quantity depending on the solvent.

Other authors [16,59,62] have used additionally chemometric tools to extract the features responsible for the differences among the analyzed samples, which were further identified with the aid of the available databases or solely using the RT plus parent ion and mass fragments. The most common chemometric tools used were PCA, PLSDA, HCA, variable importance in projection (VIP) and orthogonal projection to latent structure discriminant analysis (OPLSDA) techniques. The online databases searche here were the already mentioned ones used for the studies, not including the chemometric tools. Li et al. [59] evaluated the response to shading in *Camellia sinensis* through a well-structured data analysis with the aid of different tools. Among several compound classes identified in the leaves and shoots of two *C. sinensis* cultivars (‘Yulv’ and ‘Maotouzhong’), the authors referred to the presence of 3 anthocyanin derivatives (petunidin-3-glucoside, peonidin-3-*o*-glucoside and delphinidin-3-*o*-arabinose). Accordingly, the period of shading negatively impacted the accumulation of delphinidin-3-*o*-arabinose in the ‘Yulv’ cultivar when compared with the ‘Maotouzhong’ cultivar. Zhang et al. [16] designed a quite different study but also evaluated the impact of shade in the metabolic profile of a single *C. sinensis* cultivar. Similarly to Li et al.’s studies [59], a previous step using chemometric tools was undertaken to select metabolites responsible for the differences across the treatments. Three anthocyanin derivatives were identified (pelargonidin, cyanidin 3-(6″-caffeylglucoside) and cyanidin-3-*o*-(6″-*o*-malonyl-2″-*o*-glucoronil)), with the first two also being negatively impacted by the shade (black net and nano-insulating film versus unshaded). These were the two unique studies [16,48] comparing the shading effects in *C. sinensis* samples, from which it arose that shade negatively impacts the accumulation of anthocyanins. Quin et al. [62] also used chemometrics to extract the discriminatory features among six red-pigmented *L. sativa* cultivars and identified four cyanidin and two delphinidin derivatives with RT, parent ion and mass fragments but without using online databases. Among the identified compounds, only one (cyanidin-3-*o*-glucoside) was in common with Viacava et al.’s [63] studies in the *L. sativa* red cultivar.

Regarding the anthocyanin quantification results (Table 3), most of the published studies quantified the total anthocyanin content using spectrophotometric methods but did not perform an individual quantification process for the identified anthocyanin. From the few studies in which quantification was carried out, this task was done using high-performance liquid chromatography [20,64,72], and in the other two directly from the mass spectrometry results [69,70]. Yuan et al. [64] and McCance et al. [20] quantified the anthocyanins using HPLC– DAD at 520 nm using a standard calibration curve based on the molar concentration with authentic cyanidin-3-glucoside. It should be noted that in McCance et al.’s studies [20], significantly different anthocyanin contents were obtained for each *O. basilicum* cultivar they included and plant maturity stage they considered. Bentley and colleagues [72] used the same approach to quantify the anthocyanin compound, expressing it as malvidin-3-glucoside. Deng and colleagues [68] performed anthocyanin quantification (sweet potato leaves) using the mass spectrometry data. The authors used multiple reaction monitoring (MRM) to screen each metabolite and perform the respective quantification process. Despite this procedure, the authors did not present in the manuscript the absolute content determined for each anthocyanin derivative, but rather their relative quantity in each phase of the juvenile red fading phenomenon studied. Zhang et al. [70] also used MS data for quantification; however, these authors only performed a global semi-quantification of the anthocyanin content in *C. oblonga* leaves by means of cyanidin equivalents.

### 4.2. Carotenoids

#### 4.2.1. Samples, Pre-Processing and Extraction Details

Plant leaves for carotenoids extractions were used fresh [17,73,74], dried [18], aged [75], frozen in liquid nitrogen [76] or lyophilized [19,77,78] (Table 4). It seems that carotenoids can be extracted from leaves independently of their initial condition. However, none of the studies compared extracted carotenoids from the same sample in different starting conditions, which is a clear gap in the literature. Globally, samples were processed and analyzed immediately after harvesting. The few exceptions were those using dried [18] and aged [75] leaves. The lyophilized ones, when not processed immediately, were preceded by a freezing step [77]. Azevedo and colleagues [74] studied fresh kale leaves (*Brassica oleracea*) immediately after collection, with minimal processing. These authors compared mature and young leaves collected from both organic and conventional farms, as well as leaves from a supermarket (summer and winter), and additionally evaluated the carotenoid degradation during their shelf life (1, 2, 3, and 5 days at 7–9 °C). According to the obtained results, the carotenoid content was affected by the maturity of the leaves mostly on conventional farms, according to the season and storage time. An additional study [76] also reported that leaves from the same plant, collected simultaneously but differing in color, possessed significant differences (Duncan’s multiple range test) in carotenoid content. No other comparisons of the carotenoid contents of leaves belonging to the same species were performed in the remaining studies. Instead, comparisons among the structures of the same plant (leaves, stem, roots, rhizomes, flowers and fruits) [18,73,77], cultivars of the same species [73,76] or among different species [19,77] or transgenic or mutant plants [17,78] were performed. It arose from those studies that the carotenoid contents clearly differ among the cultivars and plant structures, being higher in the leaves when compared with the rhizomes [77], fruits and flowers [73] or with the stems and roots [18].

Regarding the solvents used for the extraction, all but two [18,76] included pure acetone [73,74,78] or acetone in a mixture [17,19,75,77]. Only two published studies compared the carotenoid contents obtained with different solvents [18,78]. Mi et al. [78] reported that the best results were achieved with methanol, even when compared with acetone (the solvent that seems to be the most popular for carotenoid extraction). These authors [78] concluded that β-cyclocitral (an apocarotenoid) was only detected in methanol extraction systems (MeOH or MeOH + 0.1% BHT), while the others were detected at significantly higher levels. The worst results were obtained with ethyl acetate or *n*-hexane, depending on the apocarotenal that was considered. In Santos et al.’s work [18], among the three tested solvents (hexane, ethyl acetate and ethanol), the worst results seemed to be highly dependent on the plant structure that was considered. These authors emphasized the relevance of the solvents in the extraction procedures, clearly stating that the polarity of the solvent affects the nature of the metabolites extracted, accounting for the very diverse chemical extract composition. Regarding the sample/solvent ratios reported here, they highly differed and sometimes were not mentioned in the Materials and Methods sections [17,18,74,75]. Among those who clearly stated the ratios, around 10–100 mg of sample was used per mL of solvent.

Regarding the extraction procedures, one seemed to be quite simplistic, being performed in a single extraction step [76] followed by a centrifugation, while the others included repeated extractions followed by evaporation and re-dissolution in different solvents [17,18,19,73,74,77,78]. Besides those laboratorial steps, Murillo et al. [75] also included a saponification step prior to the analysis. This last step, the saponification, seems to be quite controversial, with some authors [18] clearly saying that they did not perform it to avoid losses, especially of the more polar carotenoids (lutein, violaxanthin and neoxanthin). It is not clear if these highly different extraction procedures are related to the different kinds of plant species used in each of the studies, nor if they have been optimized to obtain satisfactory extraction yields for the extracted compounds in the studies, using very different plant structures such as leaves and roots. In these studies, comparisons between extraction procedures were performed or mentioned; however, it seems that repeated extractions were beneficial to the results once performed in most of the studies. ThAe comparison of the extraction procedures appears to be a literature gap, which could help to reduce the time and costs, at least for the more laborious laboratorial procedures.

#### 4.2.2. Mass Spectra Acquisition

The carotenoid extract mass spectra acquisition process was always preceded by a high- or ultrahigh-performance liquid chromatography step (Table 5). The separation step was all the times performed using reversed-phase columns, a C30 carotenoid column [17,73,75] or a C18 column [18,19,74,76,77,78] measuring (100–250) × (2.1–4.6) mm and a particle size range of 1.7–5 µm. Mi et al. [78] used a UPLC BEH C18 column in their work and an additional C18 guard column, while none of the remaining studies referred to using such instruments. These authors also mentioned that separation in this column system proved to be superior to the one achieved using a UPLC C8 or HPLC C30 column; however, no further details about it were given. Regarding the eluent(s) used for the chromatographic separation, Jayaraj and colleagues [17] used a single solvent (methanol), with the elution step being performed in an isocratic way. An elution gradient was used in all of the remaining studies, with the most common solvents being combinations of methanol, acetonitrile and water in different proportions. Saini et al. [73] and Murillo et al. [75] also referred to the use of methyl tertiary butyl ether and Mi et al. [78] used 2-propanol in significant amounts. The use of formic acid as a buffer (in about 0.1%) is also common [18,19,76,77,78]. Besides the comparison of the chromatographic columns, Mi and colleagues [78] also compared the performance of different elution systems. The authors concluded that the use of H_2_O/acetonitrile–acetonitrile.2-propanol benefits the elution of non-poplar apocarotenoids when compared with the H_2_O–acetonitrile elution system. Additonally, the solvent ratios were optimized, with the best results being achieved with acetonitrile/2-propanol (60:40) and H_2_O/acetonitrile (80:20). None of the other authors mentioned any details about possible elution systems or gradient optimization steps.

Regarding the mass spectra acquisition, it is widely known that the ionization source plays a crucial role in the whole process. With poor ionization, the success of the experiments is highly compromised. In the studies herein considered, two types of ionization were employed: atmospheric pressure chemical ionization (APCI) [17,18,73] and electrospray ionization (ESI) [19,74,75,77,78]. Azevedo et al. [74] employed what seems to be an advantageous type of ESI, thermabeam ESI. According to the manufacturer, this ionization source possesses several advantages over traditional particle beam interfaces (traditional ESI), such as decreased dispersion during desolvation, resulting in increased sensitivity; higher efficiency in solvent evaporation; and low consumption of helium due to the absence of aerosols, among others. All of the studies undertaken with ESI were performed in the positive ionization mode, as expected due to the chemical characteristics of the carotenoid molecules. No studies referred to the choice of the ionization source, with no information stating whether the selected one was the only available or if an alternative was unsuccessfully tried (some of the available apparatuses have more than a single ionization source). The mass spectrometry techniques employed in the published literature used three distinct types of mass analyzers: ion trap [17], quadrupole time-of-flight [18,19,73,74,75,77] and orbitrap [78]. As previously mentioned (Section 3), these mass analyzers show distinct performances based on the kinds of analytes, mass range, sensitivity and signal-to-noise ratio, which probably impact the results. However, due to the absence of a study comparing them, or at least different studies performed on the same plant species, it was not possible to draw conclusions about the results obtained with the different mass analyzers.

#### 4.2.3. Identified and Quantified Carotenoids

Globally, a large number of carotenoids were identified in edible plant leaves via mass spectrometry (Table 6). Some of the studies, besides the identification of a specific carotenoid, also determined its content in the plantm while others additionally compared the contentw among the different plant structures, wild- and transgenic-type leaves [17], cultivars [73], seasons, leaf maturity stages, storage times and farming systems [74].

Regarding compound identification, the published studies are quite distinct. Some of them have focused on identification via mass spectrometry experiments combined with additional chromatographic (RT) or spectrophotometric (λmax) parameters [17,19,73], while others used other laboratorial techniques, with MS being solely used for mass compound confirmation [74]. Two of the published studies also used available online databases or chemometric tools for carotenoid identification alone [18] or in combination with RT and λmax [76]. The use of standards for the compound structure confirmation was also commonly observed [74,75,77,78]. Jayaraj et al. [17] compared transgenic and wild-type carrot leaves and identified 4 carotenoids in wild-type leaves, while in transgenic lines the same 4 were identified plus an additional 6 carotenoids. According to the authors, the identification was performed using HPLC and mass spectrometry, but no additional details about it were given. Lachowich and colleagues [77] identified 9 carotenoids in two different plant species on the basis of the fragmentation patterns and PDA profiles. Parent ion, fragmentation, RT and PDA profiles (λmax) were compared with authentic standards when available, or if not available were compared with the literature data. Wojdyło et al. [19] identified 7 carotenoids in leaves from 8 fruit trees through the RT, λmax, parent ion and MS/MS fragments, with the isomers being distinguished by their λmax or specific MS/MS fragment intensity values in accordance with the previous literature. Murillo et al. [75] undoubtedly identified 19 carotenoids among 32 carotenoid-type compounds in *Zamia dressleri* leaf extracts. The identification results were based on RT, λmax, *cis* peak intensity, parent ion and MS/MS fragments when compared with authentic standards. No additional tools were used, such as online available databases, which could be of benefit in the identification of the remaining coumpounds. Mi et al. [78] identified 20 apocarotenoids (APOs) through their RT, parent ion and mass fragmentation profiles compared with authentic standards. This study was not included in Table 6 because it did not deal directly with carotenoids but instead with APOs, and also because the quantification results were presented in a graphic format, which precludes the knowledge of the exact carotenoid content information. Saini et al. [73] identified 6 carotenoids in *Moringa oleifera* leaves, flowers and tender fruits via mass spectrometry using information from previously extracted carotenoids from leaves of the same plant. According to these authors, their previously extracted carotenoids from *M. oleifera* leaves, using a well stablished protocol, could be used as standards for further identification. No commercial standards were used, nor were any other tools used besides the RT, λmax, spectral fine structure and mass spectrum data from the in-house-obtained standards for the identification. Azevedo and colleagues [74] unequivocally identified in kale leaves 7 distinct carotenoids using other laboratorial techniques, and used mass spectrometry solely for the compound mass confirmation process. According to these authors, several inconclusive or even erroneous identification results for the referenced chromophores were encountered in the literature. They proposed that to achieve precise carotenoid identification, the minimum requirements need to be fulfilled; namely, the λmax needs to be observed in two different solvents, the chromatographic properties (RT) need to be identical in two systems when compared with the corresponding standards and the mass spectrometry results should confirm the molecular mass. Azevedo et al. [74], besides fulfilling these criteria, additionally carried out chemical reactions to confirm the type and position of the molecule functional groups. Santos et al. [18] and Shen et al. [76] included the use of online databases for compound identification. Santos et al. [18] was only able to identify a carotenoid-type compound upon observing characteristic physicochemical properties obtained via MS. The exact identification of the compound was not possible through an *m/z* search in the Metlin database, which was the only approach undertaken by the authors. Shen and colleagues [76] undertook several steps for carotenoid identification among purple, intermediately purple and complete green *Camellia sinensis* leaves of the “zixin” cultivar. The first step was dedicated to the identification of discriminant compounds among the 3 types of leaves using several chemometric tools (PCA, PLSDA, VIP, FDR and HCA). Those compounds were first putatively identified based on the search of accurate masses of significant peak features against the online KEGG and HMDB databases (with LC-MS results). The putative identities were additionally confirmed using tandem mass spectrometry, giving the compounds’ exact mass values, isotopic distribution and MS/MS fragmentation spectra.

Regarding the carotenoid composition of the plant leaves, the most commonly identified carotenoids were β-carotene, lutein and zeaxanthin, which according to the literature are among the most common carotenoids found in plant leaves. It should be stressed that the studies considered in this review only included mass spectrometry studies on edible plants (many others exist based on other laboratorial techniques), preventing conclusions being drawn about the plant leaves’ carotenoid composition.

A carotenoid quantification process was performed in 6 of the published studies [17,19,73,74,77,78], although never directly through mass spectrometry. Although the quantification processes were not performed using mass spectrometry, the knowledge of these values is very relevant, allowing one to identify at least the concentration range at which it is still is possible to detect specific carotenoids using mass equipment. Some of the quantification results refer to the dry weight (dw), while others refer to the fresh weight (fw); however, from Table 6, it is clear that most carotenoid contents in plant leaves are in the range of a few µg per g of leaf. The carotenoid contents in Table 6 are expressed for each published study, in a range format including the lower and higher values quantified among all samples studied or a single value when a single sample was included.

### 4.3. Chlorophylls

#### 4.3.1. Samples, Pre-Processing and Extraction Details

As expected, chlorophylls were a class of molecules that were extensively identified and quantified in plant leaves, including edible ones. However, there are few studies in which such an analysis was performed using mass spectrometry (Table 7). Indeed, only four published studies were dedicated to the analysis of this class of compounds using this spectrometric technique [19,77,79,80].

Regarding the samples used for chlorophyll extraction, Wojdyło et al. and Lachowicz et al. [19,77] used freshly lyophilized samples. Delpino-Rius et al. [79] referred to the use of commercial unprocessed samples; however, these were probably dried samples, as they were dealing with tea leaves ready to be consumed. Kao et al. [80] performed a unique study including a comparison of samples processed in different manners; namely, dried under controlled conditions and lyophilized. Regarding the sample diversity, Lachowicz and colleagues [77] compared the rhizomes and leaves of 2 different plant species, while Wojdyło et al. [19] dedicated their studies to comparing 8 fruit plants, including 3 different cultivars per plant in two seasons. Delpino-Rius et al. and Kao et al.’s [79,80] studies were dedicated to single species, namely *C. sinensis* (including 4 cultivars) and *Rhinacanthus nasutus*, respectively. It should be stressed that studies dealing with multiples species or cultivars provide added value, as it is widely known that the samples (fresh or dried) and the corresponding extraction procedures highly impact the results.

Regarding the chlorophyll extraction, Wojdyło et al. [19] and Lachowicz et al. [77] also determined the carotenoid contents of their samples (as detailed in Section 4.2) and used the same extracts. These authors included hexane and acetone in their solvent mixtures, which seemed to not only to facilitate the carotenoid extraction but also the extraction of chlorophylls. These two organic compounds were similarly used by Kao and colleagues [80]. Globally, chlorophylls, for mass spectrometry measurements, were extracted with an acetone, hexane and alcohol (ethanol or methanol) mixture. The exception was Delpino-Rius et al.’s [79] study, in which acetone was the single solvent used. The sample/solvent ratio varied among samples in the range of 5–100 mg per mL of extraction solvent.

Regarding the extract preparation, the chlorophyll extraction seemed to require quite complex experimental procedures. All studies but one [79] undertook successive extraction steps followed by evaporation and re-dissolution in appropriate solvents (methanol or acetone). Delpino-Rius and colleagues [79] performed an extraction process that was apparently very simple, solely using 80% acetone. No additional information was given on the extraction conditions nor any experimental procedure, and nothing more was found, even when consulting the references provided by the authors in the Materials and Methods section of the paper.

#### 4.3.2. Mass Spectra Acquisition

Similarly to what was observed for the remaining pigments considered in this revision, the chlorophyll mass spectra acquisition step were always preceded by a high-performance liquid chromatographic step (Table 8). Compounds separation was undertaken in the four published studies in reversed-phase C18 columns measuring (100–150) × 2.1 mm and with a particle size range of 1.7–1.8 µm. A single study [79] compared the performances of two columns (ACQUITY UPLC BEH C18 and ACQUITY UPLC HSS T3) and selected the HSS T3 due to the higher resolution shown. However, the authors also said that the selected column increased the total analysis due to enhanced non-polar derivative retention. Chlorophyll elution was always performed in a gradient manner combining two [14,77,79] or three [80] mobile phases. Acetonitrile and methanol were always employed in different proportions, while in some cases formic acid, isopropanol or N,N-dimethylformamide was added in small portions.

Regarding the ionization source used in the chlorophyll analysis, electrospray ionization (ESI) in the positive mode was used by Wojdyło et al. [19] and Lachowicz et al. [77], while APCI was employed in Kao et al.’s [80] study. Interestingly, Delpino-Rius et al. [79] used both types of ionization sources—APCI for apolar compounds and ESI for the more polar ones. Additionally, these authors reported the use of target metabolomics (multiple reaction monitoring—MRM) after the optimization of the collision energy to confirm the compound’s identity. The information about the mass analyzers employed in the four published studies is not explicitly available for all of them. Two studies used a quadrupole time-of-flight analyzer [19,77]; however, Delpino-Rius et al. [79] and Kao et al.’s [80] studies only mentioned the use of tandem mass spectrometry without any additional information. This information is of particular relevance due to the already mentioned performance variability among instruments and should be mentioned for clearer interpretation of the results.

#### 4.3.3. Identified and Quantified Chlorophylls

Chlorophyll identification was performed in the published studies in different plant species [19,77], cultivars [19,79], structures [77], seasons [19] and sample processing conditions [80]. A list of the identified and quantified compounds can be found in Table 9. Lachowich et al. [77] based the chlorophyll identification process on their RT, λmax, parent ion and MS/MS fragments. These authors identified 12 chlorophys (chorophyll, chlorophyllide and pheophytin isomers) in the leaves and rhizomes of both species. Other authors [19] also identified 16 distinct chlorophylls (chlorophyllide, pheophorbid and pheophytin isomers) in 8 different fruit tree leaves based on their RT, λmax, parent ion and MS/MS fragments. The authors additionally compared each fruit tree and the results obtained over two seasons (spring and autumn) and for 2 or 3 cultivars of each tree. Delpino-Rius et al. [79] performed a quite complete chlorophyll screening process and identified 25 chlorophylls through their RT, λmax, S/Q ratio (absorbance of Soret (S) and Q-bands (Q)), parent ion and MS/MS fragments. The same parameters were used by Kao and colleagues [80] to identify 12 chlorophylls in *R. nasatus* leaves processed in two different ways (hot-air-dried and freeze-dried). Interestingly, in freeze-dried samplesm only 4 of the 12 compounds were identified.

Similarly, to what was observed for anthocyannins and carotenoids, chlorophyll quantification was never performed using mass spectrometry. Quantification results were always obtained through calibration curves obtained from the liquid chromatography results. Lachowich and colleagues [77] used standards to obtain calibration curves and to further quantify chlorophylls a and b, chlorophyllide b and pheophytins a and b, while hydroxypheophytins a and b and pheophytins a’ and b’ were expressed as pheophytins a and b and hydroxychlorophylls a, and chlorophylls a’ and b’ were expressed as chlorophylls a and b. The authors stated that the chlorophyll content of the leaves was 2 times higher than in rhyzomes, which was an expected result. Regarding the two plant species, besides being of the same genus, *F. japonica* possesses a much higher content (about 1.5 times) of all identified compounds, with the most abundant chlorophyll being pheophytin b, followed by chlorophylls b and a for both species. Wojdyło et al. [19] also quantified their chlorophylls in a similar manner, obtaining calibration curves for chlorophylls a and b, pheophytin a and pheophorbid a. The results presented in Table 9 correspond to the range of values encountered in the analyzed samples; however, some of the compounds were not detected in all of the analyzed samples. For example, hydroxychlorophyll b, chlorophyllide a and hydroxypheophitin b were never detected in any of the cultivars of sweet cherry, plum, apricot or quince, nor in any season. Regarding the concentrations range, very different chlorophyll contents were present in each species. Pheophytin (b + b’) was also the pigment that reached the highest concentration of 311.3 ± 3.1 g/100 g (dw) in “harcot” apricot spring leaves. In Delpino-Rius et al.’s [79] study, chlorophyll quantification was undertaken using commercially available standards and their derivatives. Data for the lower and higher concentrations obtained are presented in Table 9. A pheophytin isomer was also the compound that achieved the highest concentration, followed by chlorophyll b. Regarding the comparison between commercial and luxury teas, the authors concluded that luxury teas possess higher chlorophyll contents, which was an a priori expected result due to the less aggressive treatment during the manufacturing process. Kao and co-workers [80] quantified 12 chlorophylls through the corresponding standard or derivative calibration curves, including an internal standard. The authors reported several differences among the chlorophyll contents of hot-air-dried and freeze-dried samples, with the latter being richer in chlorophylls a and b and poorer in chlorophyll a’ and pheophitin a. Interestingly, the remaining chlorophylls (presented in Table 3) were not detected in freeze-dried samples. Globally, considering the four studies, the chlorophyll content range of green leaves varied from a few µg to several mg per g of leaf, with *C. sinensis* being the species that seems to possess the highest content of this compound class.

## 5. Critical Analysis and Conclusions

Natural pigments included in this review (anthocyanins, carotenoids and chlorophylls) are the target of many published studies, mostly after the year 2000, clearly in parallel with the emergence of the OMICS concept. All of the published studies focused on different plant species, with nearly 30 in total. The exceptions were observed for *C. sinensis* (7 studies: 5 anthocyanins, 1 carotenoid and 1 chlorophyll), *L. sativa* (2 studies: anthocyanins) and *B. oleracea* (2 studies: 1 anthocyanins and 1 carotenoids), for which more than one study was encountered. Regarding the samples employed, they were used fresh, dried, frozen in liquid nitrogen or lyophilized. Pigment extraction rocesses were mostly performed with well-known organic solvents such as methanol, ethanol (manly anthocyanins) or acetone and hexane (carotenoids and chlorophylls). A unique study was encountered using quite unusual solvents, namely natural deep eutectic solvents (NaDES), for anthocyanins extraction [72]. The extraction conditions (time, temperature or number and complexity of the experimental steps) were very different for each class of pigments, but also among the studies dedicated to the same pigment type. All of the studies but one [71] employed a previous chromatographic separation step before the MS experiments. Chromatographic separation steps were mostly undertaken using reversed-phase C18 columns (all pigments classes), less commonly with a C30 column (carotenoids) [17,73,75] and in a single study with a C8 column (anthocyanins) [67], always through an elution gradient. Regarding the MS experiments, the mostly used ionizations source was ESI (in positive and negatives modes), with APCI being employed uniquely in three studies dedicated to carotenoid characterization [17,73,75] and in one analyzing chlorophylls [80]. A single study [79] used both types of ionization source, depending on the polarity of the compounds under analysis (APCI for apolar compounds and ESI for polar ones). Regarding the plant pigment composition, more than 70 anthocyanins, 50 carotenoids and 30 chlorophylls were identified. Studies employing chemometric tools and available online databases were generally able to clarify the chemical structures of the extracted pigments.

From the published literature, it appears that more systematic pigment extraction procedures, at least for the same pigment class, could positively contribute to comparisons across species. Additionally, the use of chemometrics combined with available databases could add value to these kinds of studies.

## Figures and Tables

**Table 1:** Anthocyanins—sample pre-processing and extraction details.

Plant	Sample	Solvents	Sample:Solvent	Time Extraction/Conditions	Ref
*C. sinensis*	Commercial tea leaves	MeOH (0.1% TFA)	250 g:no info	Maceration (overnight, 4 °C)—complex procedure with the isolation of 4 structures	[58]
*C. sinensis*	Frozen in N_2_	Cold MeOH	100 mg:1000 mL	Chloroform+H_2_O (vortex 1’)—centrifuge (4000 rpm, 15’)	[59]
*C. sinensis*	Frozen in N_2_-lyophilized	MeOH:H_2_O:FA (75:24:1)	0.1 g:1 mL	Ultrasonic bath (10’)—centrifuge (12,000 rpm, 10’)	[16]
*C. sinensis*	Frozen in N_2_-lyophilized	MeOH:H_2_O:FA (75:24:1)	25 mg:1 mL	Ultrasonic bath (15’)— centrifuge	[60]
*C. sinensis*	Stored (−80 °C)-lyophilized	MeOH (0.1 mg L^−1^ lidocaine)	100 mg:1 mL	Overnight (4 °C)—centrifuge (10’; 10,000× *g*)	[61]
*L. sativa*	Lyophilized	MeOH:H_2_O:FA (80:19:1)	1 g:10 mL	Ultrasonic bath (12 kHz, 70′, 45 °C)	[62]
*L. sativa*	Lyophilized	MeOH:H_2_O:AA (30:65:5) + 2 g/L AscA	0.1 g:5 mL	Ultrasonic bath (10′)—centrifuge (6000 rpm, 15′, 4 °C)	[63]
*L. japonica*	Lyophilized	MeOH:H_2_O:FA:TFA (70:27:2:1)	500 mg:10 mL	Room temp (24 h)	[64]
*P. minus*	Lyophilized	MeOH	500 mg:5 mL	1 h (room temp; 3 times)	[65]
*F. chiloensis*	Lyophilized	MeOH:FA (99:1)	5 g:50 mL	1 h (room temp; 3 times), evaporation to dryness, H_2_O-Amberlite column, eluate evaporated to dryness with redissolution of MeOH:FA	[66]
‘Mexican lime’	Frozen in N_2_	Acetone (80%) + ethyl acetate	100 mg:400 + 240 mL	Dark (ice,10’; twice), H_2_O, centrifuge (8500× *g*, 5’,4 °C)	[67]
*I. batatas*	Frozen in N_2_	MeOH	0.5 g:5mL	24 h darkness (4 °C)	[68]
*O. basilicum*	Frozen in N_2_	MeOH (0.2 M HCL)	‘known mass’:500 mL	Shaken (room temp, 40′), centrifuge (13,200 rpm; 30′), dried 3 times and reconstituted in 0.1% FA	[20]
*M. oleifera*	Dried in shade	EtOH:H_2_O (1:1)	1 g:7 mL	Ultrasonic bath (20′; 60 Hz), centrifuge (419 g; 10′)	[69]
*C. oblonga*	Dried in shade	MeOH	100 g:1000 mL	Ultrasonic bath (45′, 50 °C), evaporation dryness	[70]
*Z. mays*	Dried	MeOH:HCl 1N (85:15)	1 g:24 mL	Ice (15′)-centrifuge(3000 rpm, 5′)-redissoluion	[71]
*M. flabellifolia*	Naturally desiccated	4 different NaDES	50 mg:1 mL	Diluted H_2_O ultrasound bath (1.5 h, 50–55 °C), diluted with H_2_O centrifuge (16,000 rpm, 20′)	[72]

MeOH—methanol: EtOH—ethanol; AA—acetic acid; AscA—ascorbic acid; FA—formic acid; TFA—trifluoracetic acid; NaDES—natural deep eutectic solvents.

**Table 2:** Anthocyanins—chromatographic and mass spectrometry details.

Plant	Column	Eluent	Ionization Source	(LC)-MS Technique	Ref
*C. sinensis*	RP C18 column (250 × 4.6mm, 5 µm)	H_2_O:ACN (90:10; 0.1% TFA)—H_2_O:ACN (50:50; 0.1% TFA)	ESI +	LC-ESI-MS	[58]
*C. sinensis*	No info	H_2_O (0.1% FA)—ACN (0.1% FA)	ESI +	UPLC-MS	[59]
*C. sinensis*	RP ACQUITY UPLC HSS T3 C18 (100 × 2.1 mm, 1.8 µm)	H_2_O (0.1% FA—ACN (0.1% FA)	ESI +	UPLC-QTOF-MS	[16]
*C. sinensis*	RP Luna C18 Phenomenex (150 × 2.0 mm, 3 µm)	H_2_O—ACN (0.1% FA)	ESI −	HPLC-Orbitrap-MS	[60]
*C. sinensis*	RP ACQUITY UPLC HSS T3 C18 (100 × 2.1 mm, 1.8 µm)	H_2_O (0.04% AA)—ACN (0.04% AA)	ESI +	HPLC- ion trap-LC-MS/MS	[61]
*L. sativa*	No info	H_2_O (0.01% FA)—ACN	ESI + and −	UPLC-QTOF MS	[62]
*L. sativa*	RP ACQUITY UPLC BEH C18 column (100 × 2.1 mm, 1.7 µm)	H_2_O (0.1% AA)—MeOH (0.1% AA)	ESI + and −	UPLC-DAD-ESI-QTOF/MS	[63]
*L. japonica*	RP Zorbax Eclipse XDB-C18 (250 × 4.6 mm, 5μm)	ACN:FA(99:1)—H_2_O:FA (1:99:1)	ESI +	HPLC-DAD-ESI-MS/MS	[64]
*P. minus*	RP C18 (250 × 2.0 mm, 5 µm)	H_2_O (0.1% FA)—ACN	ESI +	LC-TOF (micrOTOF-Q) MS	[65]
*F. chiloensis*	RP Luna C18 (250 × 4.6 mm, 5 µm)	H_2_O (1% FA)—ACN	ESI + and −	HPLC-DAD-ESI-MS	[66]
‘Mexican lime’	RP Waters XBridge C8 (4.6 × 100 mm)	ACN:H_2_O (19:90, 0.5% FA)—ACN	ESI +	HPLC-MS	[67]
*I. batatas*	No info	No info	No info	UPLC-MS/MS	[68]
*O. basilicum*	nanotip in-house packed C18 (50 × 2.1 mm, 3.5 µm)	H_2_O (0.1% FA + 0.2% ACN)—ACN	ESI +	HPLC-QTOF MS	[20]
*M. oleifera*	RP Denali C18 (150 × 2.1 mm, 3 µm)	H_2_O (0.2% FA)—ACN	ESI −	HPLC-MS	[69]
*C. oblonga*	RP Zorbax eclipse plus C18 column (50 × 2.1 mm, 1.8 µm)	H_2_O (0.1% FA)—ACN (0.1% FA)	ESI +	UHPLC-QTOF-MS	[70]
*Z. mays*	No column	-	MALDI	TOF MS	[71]
*M. flabellifolia*	RP ACQUITY BEH C18 column (100 × 2.1 mm, 1.7 µm)	H_2_O (0.1% FA)—ACN (0.1% FA)	ESI −	UPLC QTOF MS	[72]

RP—reversed-phase; ACN—acetonitrile; MeOH—methanol; TFA—trifluoroacetic acid; FA—formic acid; AA—acetic acid; ESI—electrospray ionization in positive (+) or negative (−) mode; MALDI—matrix-assisted laser desorption ionization; MS—mass spectrometry; LC—liquid chromatography; HPLC—high-performance liquid chromatography; UHPLC—ultrahigh-performance liquid chromatography; TQ—triple quadrupole; DAD—diode array detection; QTOF–quadrupole time-of-flight.

**Table 3:** Anthocyanins identified or quantified in edible plant leaves.

	[58]	[59]	[16]	[60]	[61]	[62]	[63]	[64] ^#^	[65]	[66]	[67]	[68]	[20] ^#^	[69]	[70]	[71]	[72]
Cyanidin (Cyn)									♦						♦	♦	
Delphinidin (Delp)																	
Malvidin (Mal)																♦	
Pelargonidin (Pel)			♦													♦	
Peonidin (Peo)																♦	
Petunidin (Pet)																	
Cyn-3-(acetyl)-gluc								1.08 ± 0.04									
Cyn-3-*o*-(6″-*o*-acetyl)-gluc							♦										
Cyn-3-acetyl glucosamine																	♦♦
Cyn 3-(6″-caffeylgluc)			♦														
Cyn 3-*p*-coumaroyl gluc																	♦♦
Cyn-3-(*p*-coumaroyl)-rutin-5-gluc								0.89 ± 0.04									
Cyn 3-(*p*-coumaroyl) derivative											♦						
Cyn-3-*o*-(6-*p*-coumaroyl-60-caffeoyl)sophoroside-5- *o*-gluc													0.13 ± 0.02–0.82 ± 0.23 ^##^				
Cyn-3-*o*-(6-*p*-coumaroyl-X-malonyl-60-caffeoyl)sophoroside-5-*o*-glucoside *													0.05 ± 0.01–0.44 ± 0.11 ^##^				
Cyn-3-*o*-(6,60-di-*p*-coumaroyl)sophoroside-5-*o*-gluc													0.41 ± 0.02–2.02 ± 0.55 ^##^				
Cyn-3-*o*- (6,60- di-*p*-coumaroyl-X-malonyl)sophoroside-5-*o*-gluc *													0.10 ± 0.01–1.18 ± 0.31 ^##^				
Cyn-3-*o*-β-D-(6-(E)-*p*-coumaroyl) galpyr	♦																
Cyn 3-(*p*-feruloyl) derivative											♦						
Cyn 3-galact					♦			0.37 ± 0.02									
Cyn-3-*o*-β-D-galact	♦																
Cyn-3-*o*-gluc					♦	♦	♦	32.74 ± 0.71				♦♦			♦		
Cyn-3-*o*-gluc chloride					♦												
Cyn 3-*o*-glucosyl-magluc						♦											
Cyn-3,5-*o*-digluc						♦		39.13 ± 0.87				♦♦		♦			
Cyn-3-digluc-5-gluc								1.56 ± 0.04									
Cyn *o*-hexosyl-*o*-hexosyl-*o*-hexoside					♦												
Cyn 3-malgluc **											♦						
Cyn 3-(3″-malgluc)									♦								
Cyn-3-*o*-(3″-*o*-malonyl)-gluc							♦										
Cyn-3-*o*-(6″-*o*-malonyl)-gluc							♦										
Cyn-3-*o*-(6″-*o*-malonyl-2’’-*o*-glucoronil) gluc			♦														
Cyn-*o*-malonyl-malonylhexoside						♦											
Cyn 3-rutin								1.27 ± 0.05			♦						
Cyn 3-rutin-5-gluc								4.18 ± 0.13									
Cyn 3-*o*-sophoroside											♦						
Cyn *o*-syringic acid					♦												
Cyn 3-*o*-[2″-*o*-(xylosyl)-6″-*o*-(*p*-*o*-(glucosyl)-*p*-coumaryl)gluc]5-*o*-gluc									♦								
Delp 3-*o*-arabinose		♦															
Delp 3-coumaroyl gluc																	♦♦
Delp 3- *o*-β-D-(6-(E)-*p*-coumaroyl) galpyr	♦																
Delp 3-galac															♦		
Delp 3-*o*-β-D-galac	♦																
Delp gluc																♦	
Delp 3-gluc																	♦♦
Delp 3-*o*-gluc						♦					♦						
Delp hexose-coumaroyl				♦													
Delp 3-(6″-malonylgluc)/6-OH-cyn 3-(6-malonylgluc)									♦								
6-OH-delp-3-(6-malonylgluc)									♦								
Delp derivative											♦						
Mal-3-acetyl gluc																	♦♦
Mal-3-coumaroyl gluc																	♦♦
Mal-3-gluc																	♦♦
Mal 3-*o*-gluc						♦											
Pel gluc																♦	
Pel 3-(6″-*p*-coumsambubi)-5-(6″-magluc)									♦								
Peo 3-*o*-(6″-acetyl-gluc)														♦			
Peo 3-*o*-gluc		♦										♦♦					
Peo 3-*o*-hexoside												♦♦					
Peo 3-malgluc											♦						
Peo 3-rutin									♦								
Peo 3-*o*-sophoroside-5-*o*-gluc												♦♦					
Pet 3-acetyl gluc																	♦♦
Pet 3-coumaroyl gluc																	♦♦
Pet 3-gluc		♦															
Pet gluc																♦	
Procyn tetramer ***										♦							
Proanthocyn III				♦													
Prodelp-*O*-gallate II				♦													

Compounds identifyed (♦) and quantified (♦♦) but no absolute quantities reported or data were in a graphical format preventing the extraction of the exact values. Gluc—glucoside; galact—galactoside; rutin—rutinoside; galpyr—galactopyranoside; coumsambubi—coumarylsambubioside; malgluc—malonyglucoside * The X denotes that the position of the malonyl substituent could not be unambiguously identified. ** The authors reported two different isomers. *** The authors reported several procyanidin tetramers with distinct masses at different retention times but were not able to unequivocally identify them. ^#^ Expressed as cyn-3-gluc Eq mg/100 g FW. ^##^ Range of values corresponding to the higher and lower contents determined for the three cultivars at the different maturity stages.

**Table 4:** Carotenoids—sample pre-processing and extraction details.

Plant	Sample	Solvents	Sample:Solvent	Time Extraction/Conditions	Ref
Carrot leaves	Fresh leaves	Hexane:Acetone:EtOH:Toluene (10:7:6:7)	1 g: no info	Extraction (56 °C)-mix with 10% sodium sulphate (epiphase withdrawn),evaporation to dryness, dissolution in chloroform	[17]
*M. oleifera*	Fresh leaves	Cold acetone	5 g: 50–100 mL	repeated extractions, partition to 10% ethyl ether in PE, evaporation to dryness, dissolution in acetone	[73]
*B. oleracea*	Fresh leaves ground in household food processor	Cold acetone	3–5 g:no info	Extraction-partition to 10% ethyl ether in PE, evaporation to dryness, dissolution in acetone	[74]
*R. communis*	Dried (25 °C) and powdered	Hexane; EtOH and ethylacetate	No info	Maceration-ethyl acetate and EtOH extraction, evaporation to dryness, dissolution in ACN	[18]
*Z. dressleri*	Brown 20 days old leaves	Acetone + 10 g NaHCO_3_	100 g:no info	Repeated extractions till colorless samples-extract diluted in ether:hexane (1:1), washed (H_2_O), dried (Na_2_SO_4_), evaporation to dryness, saponification	[75]
*C. sinensis*	Frozen in liquid N_2_ (stored −80 °C)	Cold MeOH:H_2_O (1:1)	25 mg:800 µL	TissueLyser LT (5′, 60 Hz)-, centrifuged (20′, 25,000× *g*, 4 °C)	[76]
*F. japonica//* *F. sachalinensis*	Frozen (−25 °C)-lyophilized (stored −80 °C)	Hexane:Acetone:MeOH (2:1:1) (10% of MgCO_3_ in BHT–1%)	500 mg:5 mL	Orbital shaker (300 rpm, 30′) dark—centrifuged (10′, 19,000× *g*, 4 °C), re-extraction, evaporation to dryness, dissolution in MeOH	[77]
*S. oleracea*	Lyophilized-powdered (stored at −20 °C)	*n*-hexane; Dichloromethane; Ethyl acetate; Acetone; MeOH; MeOH (0.1% BHT) **	20 mg:2 mL	15’ ultrasound, centrifuged (8′, 3800 rpm, 4 °C), re-extraction, evaporation to dryness—dissolution in ACN	[78]
Fruit tree leaves *	Lyophilized-powdered	Hexane:Acetone:MeOH (2:1:1) (10% of MgCO_3_ in BHT–1%)	100 mg:3 mL	Orbital shaker (300 rpm, 30′), dark—re-extraction 4 times, evaporation to dryness, dissolution in MeOH	[19]

* Apple, pears, quince, apricot, peach, plums, sweet and sour cherry. ** Solvents tested. Best results obtained with MeOH (0.1% BHT). Results only reported for MeOH (0.1% BHT). MeOH—methanol; EtOH—ethanol; PE—petroleum ether; BHT—butylated hydroxytoluene; ACN—acetonitrile.

**Table 5:** Carotenoids—chromatography and mass spectrometry details.

Plant	Column	Eluent	Ionization Source	(LC)-MS Technique	Ref
Carrot leaves	RP YMC C30 carotenoid column	MeOH-MTBE (0–100%)	APCI	HPLC Quadrupole Ion trap MS	[17]
*M. oleifera*	RP YMC C30 carotenoid column (250 × 4.6 mm, 5 µm)	MeOH:MTBE:H_2_O (81:15:4)–MTBE/MeOH (91:9)	APCI	HPLC-QTOF MS	[73]
*B. oleracea*	RP C18 Spherisorb ODS2 (150 × 4.6 mm, 3 µm)	CAN (0.05% triethylamine):MeOH:ethyl acetate (95:5:0 to 60:20:20)	Thermabeam ESI +	HPLC MS	[74]
*R. communis*	RP C18 column (3.0 × 150 mm, 2.6 µm)	H_2_O (0.1% FA)–ACN (0.1% FA)	ESI +	LC-microTOF MS	[18]
*Z. dressleri*	RP YMC C30 carotenoid column (250 × 4.6 mm, 3 µm)	MeOH:MTBE:H_2_O (81:15:4)–MeOH:MTBE:H_2_O (6:90:4)	APCI	QTOF LC MS	[75]
*C. sinensis*	RP ACQUITY UPLC BEH C18 column (100 × 2.1 mm, 1.7 µm)	H_2_O (0.1% FA)–ACN (0.1% FA)	ESI+/ESI−	UPLC-QTOF MS	[76]
*F. japonica//* *F. sachalinensis*	RP ACQUITY UPLC BEH C18 column (100 × 2.1 mm, 1.7 µm)	ACN:MeOH (7:3)–H_2_O (0.1% FA)	ESI +	LC-QTOF MS	[77]
*S. oleracea*	RP ACQUITY UPLC BEH C18 column (100 × 2.1 mm, 1.7 µm) + UPLC BEH C18 guard column (5 × 2.1 mm, 1.7 µm)	H_2_O:ACN:FA (80:20:0.1)–ACN:IPA:FA (60:40:0.1)	ESI +	UHPLC-Q-Orbitrap MS	[78]
Fruit tree leaves *	RP ACQUITY UPLC BEH C18 column (100 × 2.1 mm, 1.7 µm)	H_2_O (0.1% FA)–ACN:MeOH (7:3)	ESI +	LC-PDA-QTOF MS	[19]

* Apple, pears, quince, apricot, peach, plums, sweet and sour cherry. RP—reversed-phase. MeOH—metanol; MTBE—methyl tertiary butyl ether; ACN—acetonitrile; FA—formic acid; IPA—2-propanol; APCI—atmospheric pressure chemical ionization; ESI—electrospray ionization in positive (+) or negative (-) mode; MS—mass spectrometry; LC—liquid chromatography; HPLC—high-performance liquid chromatography; UHPLC—ultrahigh-performance liquid chromatography; TQ—triple quadrupole; PDA—photodiode; QTOF—quadrupole time-of-flight.

**Table 6:** Carotenoids and metabolites from the carotenoid pathways identified and quantified in edible plant leaves.

	Carrot Leaves (Wild //Trangenic)[17] *	*M. oleífera* [73]	*B. olerácea* [74] *	*R. communis* [18]	*Z. dressleri* [75]	*C. sinensis* [76]	*F. japonica//F. Sachalinensis* [77]	Fruit Tree [19] **
	1	2	3	4	5	6	7	9
α-carotene	20.1 ± 0.58//12.6 ± 0.62				♦			
*β*-carotene	21.8 ± 1.15//27.1 ± 0.77		30.7–42.4 ^##^		♦			♦♦
*β*-carotene-5,6-epoxide					♦			
15-*Z*-*β*-carotene		0.40–0.69						
All-*E*-*β*-carotene		11.86–20.77						
All-*trans*-*β*-carotene							63.70 ± 0.13//41.41 ± 0.25	
9-*cis*-*β*-carotene			♦^#^				19.08 ± 0.04//12.40 ± 0.07	♦♦
13-*cis*-*β*-carotene			♦^#^				4.43 ± 0.01//2.88 ± 0.02	
13-*Z*-*β*-carotene					♦			
9-*Z*-*β*-carotene					♦			
Adonirubin	5.2 ± 0.27 ^§^							
Adonixanthin	5.0 ± 0.36 ^§^							
Antheraxanthin					♦			
Astaxanthin	32.4 ± 1.5 ^§^							
Canthaxanthin	4.1 ± 0.21 ^§^					♦		
*β*-cryptoxanthin	2.8 ± 0.42 ^§^				♦			
*α*-cryptoxanthin					♦			
Lutein	68.5 ± 0.87//46.0 ± 1.28		44.0–56.7 ^##^		♦			45.2 ± 2.1–426.5 ± 4.2
lutein-5,6-epoxide							0.84 ± 0.00//0.55 ± 0.00	
All-*trans*-lutein							24.08 ± 0.05//15.65 ± 0.09	
All-*E*-lutein		17.6–41.16						
13-*Z*-lutein		1.58–5.80						
All-*E*-luteoxanthin		2.58–5.68						
Zeaxanthin	37.7 ± 0.59//29.8 ± 1.01		♦ ^#^		♦			3.1 ± 0.6–212.3 ± 2.5
All-*E*-zeaxanthin		2.26–13.54						
All-*trans*-zeaxanthin							4.46 ± 0.01//2.90 ± 0.02	
Violaxanthin			29.2–42.2 ^##^					
*trans*-violxanthin							0.68 ± 0.00//0.44 ± 0.01	
9-*cis*-violaxanthin								0.3 ± 0.0–8.4 ± 1.1
Neoxanthin			12.0–25.9 ^##^		♦			
9-*cis*-neoxanthin								1.3 ± 0.1–33.6 ± 1.1
9-Z′-neoxanthin					♦			
Capsanthin					♦			
13/13-′*Z*-capsanthin					♦			
Capsoneoxanthin					♦			
Capsorubin					♦			
13-*Z*-capsorubin					♦			
Cryptocapsin					♦			
Cryptocapsin 5,6-epoxide					♦			
9-*cis*-*β*-cryptoxanthin								11.0 ± 1.7–504.5 ± 4.5
Carotenoid compound				♦♦♦				
4,4′-diapolycopenedial						♦		
3,4-dihydroanydrorhodovibrin						♦		
3′,4′-dihydrorhodovibrin						♦		
OH-spheroidene						♦		
Echinenone	2.4 ± 0.31^§^							
3′-OH-echinenone						♦		
Isorenieratene						♦		
Presqualene diphosphate						♦		
8′-*R*-neochrome							1.91 ± 0.00//1.24 ± 0.01	
8′-*S*-neochrome							2.31 ± 0.00//1.50 ± 0.01	

* Values presented per gram of fresh weight (FW). The remaining values qre presented per gram of dry weight (DW). ** Apple, pears, quince, apricot, peach, plums, sweet and sour cherry. Presented values corresponds to the lower and higher contents determined among all samples. ^§^ Solely encountered on trangenic leaves. Carotenoids identified ^#^ and carotenoids identified and quantified ^##^ using additional techniques. ♦ Carotenoids identified but not quantified. Note: ♦♦-carotene and 9-cis-carotene were quantified together: 13.3 ± 1.1–669.1 ± 3.7 *g/g* (dw). ♦♦♦ Carotenoid-type compound without precise identification.

**Table 7:** Chlorophylls—sample pre-processing and extraction details.

Plant	Sample	Solvents	Sample:Solvent	Time Extraction/Conditions	Ref
Fruit tree leaves *	Lyophilized, powdered	Hexane/Acetone/MeOH (2:1:1) (10% of MgCO_3_ in BHT–1%)	100 mg:3 mL	Orbital shaker (300rpm, 30′) dark—re-extraction 4 times, evaporation to dryness, dissolution in MeOH	[19]
*F. japonica//* *F. sachalinensis*	Frozen (−25 °C), lyophilized (stored −80 °C)	Hexane:Acetone:MeOH (2:1:1) (10% of MgCO_3_ in BHT–1%)	500 mg:5 mL	Orbital shaker (300 rpm, 30′) dark—centrifuged (10′, 19,000× *g*, 4 °C), re-extraction, evaporation to dryness, dissolution in MeOH	[77]
*C. sinensis* **	Unprocessed samples	Cold acetone (80%)	10 mg:2 mL	No info	[79]
*R. nasutus*	Hot air drying (60 °C, 4 h); freeze-dried; stored −20 °C	Hexane:EtOH/acetone/toluene (10:6:7:7)	200 mg:30 mL	Shake 1 h, 15 mL hexane (shake 10′), 15 mL of 10% anhydrous sodium sulphate (shake 1′), organic layer extracted (4 times 15 mL hexane), evaporation to dryness, dissolution in acetone	[80]

* Apple, pears, quince, apricot, peach, plums, sweet and sour cherry. ** Commercial white tea; “silver needle” white tea; commercial green tea; “sencha” green tea; “matcha” green tea; commercial black tea; “Kenya” black tea; commercial Pu-erh tea; “Pu-erh”. MeOH—methanol; EtOH—ethanol; BHT–butylated hydroxytoluene; ACN —acetonitrile.

**Table 8:** Chlorophylls—chromatography and mass spectrometry details.

Plant	Column	Eluent	Ionization Source	Mass Analysers	Ref
Fruit tree leaves *	RP ACQUITY UPLC BEH C18 (100 × 2.1 mm, 1.7 µm)	H_2_O (0.1% FA)–ACN:MeOH (7:3)	ESI +	LC-PDA-QTOF MS	[19]
*F. japonica//* *F. sachalinensis*	RP ACQUITY UPLC BEH C18 (100 × 2.1 mm, 1.7 µm)	ACN:MeOH (7:3)–H_2_O (0.1% FA)	ESI +	LC-QTOF MS	[77]
*C. sinensis* **	RP BEH C18 (150 × 2.1 mm, 1.7 m) and RP ACQUITY UPLC HSS T3 (100 × 2.1 mm, 1.8 µm)	MeOH:iPrOH:ACN (10:15:75)–MeOH:ACN:H_2_O (CH_3_COONH_4_, 10 mM) (25:25:50)	APCI (apolar compounds) ESI + (polar compounds)	UHPLC tandem MS	[79]
*R. nasutus*	RP Eclipse XDB-C18	MeOH:DMF (97:3)–ACN–Acetone	APCI	HPLC-DAD MS	[80]

* Apple, pears, quince, apricot, peach, plums, sweet and sour cherry. ** Commercial white tea; “silver needle” white tea; commercial green tea; “sencha” green tea; “matcha” green tea; commercial black tea; “Kenya” black tea; commercial Pu-erh tea; “Pu-erh”. RP—reversed-phase; MeOH—methanol; ACN—acetonitrile; FA—formic acid; iPrOH—isopropanol; DMF—N,N-dimethylformamide; APCI—atmospheric pressure chemical ionization; ESI—electrospray ionization in positive (+)mode; MS—mass spectrometry; LC—liquid chromatography; HPLC—high-performance liquid chromatography; UHPLC—ultrahigh-performance liquid chromatography; PDA—photodiode; DAD—diode array detection; QTOF—quadrupole time-of-flight.

**Table 9:** Chlorophylls and their derivatives identified and quantified (mg/100 g dm) in edible plant leaves.

	Fruit Tree [19] *	*F. japonica//F. Sachalinensis* [77]	*C. sinensis* [79] **	*R. nasutus* (Hot-Air//Freeze Drying) [80]
Chlorophyll a	0.1 ± 0.0–186.4 ± 2.5 ^#^	22.91 ± 0.05//14.89 ± 0.09	72 ± 3–1250 ± 30	814.1 ± 11.82//4707 ± 59 ^##^
Chlorophyll a’	^#^	1.00 ± 0.01//0.65 ± 0.00	90 ± 5–273.8 ± 1.2	131.2 ± 2.10//53.47 ± 1.30 ^##^
Chlorophyll b	7.0 ± 0.5–80.4 ± 2.6 ^#^	63.19 ± 0.13//41.07 ± 0.24	50.6 ± 0.3–1300 ± 18	324.7 ± 8.83//1280 ± 17 ^##^
Chlorophyll b’	^#^	4.45 ± 0.01//2.89 ± 0.02	30.7 ± 2.1–410 ± 6	67.08 ± 1.31//ND ^##^
Chlorophyllide a	0.1 ± 0.0–12.7 ± 1.2	1.52 ± 0.00//0.99 ± 0.01	76-6 ± 2.1–136 ± 8	
Chlorophyllide a’			87 ± 6	
Chlorophyllide b		8.91 ± 0.02//5.79 ± 0.03	70.5 ± 2.4–123 ± 16	
Chlorophyllide b’			85 ± 3–129 ± 13	
Pheophorbid a	0.3 ± 0.0–40.4 ± 3.1 ^#^		219 ± 30–1260 ± 120	
Pheophorbid a’	^#^		68.7 ±2.5–295 ± 5	
Pheophorbid b	0.2 ± 0.0–165.1 ± 0.3 ^#^		72 ± 7–321 ± 30	
Pheophorbid b’	^#^		52.0 ± 1.8–219.1 ± 1.8	
Pheophytin a	3.3 ± 0.2–221.2 ± 2.5 ^#^	1.48 ± 0.01//0.96 ± 0.01	500 ± 50–3200 ± 320	440.2 ± 7.02 // 84.07 ± 1.73 ^##^
Pheophytin a’	^#^	0.68 ± 0.01//0.44 ± 0.00	96 ± 10–573 ± 40	69.68 ± 1.15//ND ^##^
Pheophytin b	4.8 ± 0.2–311.3 ± 3.1 ^#^	75.13 ± 0.15//48.83 ± 0.29	61.1 ± 0.8–368 ± 18	39.65 ± 2.01//ND^##^
Pheophytin b’	^#^	11.51 ± 0.02//7.48 ± 0.04	58.4 ± 1.9–106 ± 10	
OH-chlorophyll a		2.34 ± 0.02//1.52 ± 0.01		206.4 ± 3.44//ND ^##^
13-OH-chlorophyll a			111 ± 5	
15-OH-lactone chlorophyll a				9.25 ± 0.45//ND ^##^
OH-chlorophyll b	0.2 ± 0.0–9.2 ± 0.3			108.6 ± 1.58//ND ^##^
13-OH-chlorophyll b			42 ± 3–226 ± 8	
OH-pheophytin a	0.3 ± 0.0–25.3 ± 0.4			88.29 ± 2.42//ND ^##^
13-OH-pheophitin a			83 ± 13–470 ± 3	
15’-OH-lactone pheophytin a			69 ± 6–222 ± 2	
Pyropheophytin a			73 ± 6–327 ± 30	
OH-pheophytin a’				69.6 ± 2.70//ND ^##^
13-OH-pheophitin a’			68 ± 3–391 ± 24	
OH-pheophytin b	2.7 ± 0.3–91.3 ± 1.7	21.29 ± 0.04//13.84 ± 0.08		
13-OH-pheophytin b			52.5 ± 4–167 ± 11	
15’-OH-lactone pheophytin b			41 ± 4–114 ± 11	
13-OH-pheophytin b’			36.1 ± 2.3–159 ± 2	

* Apple, pears, quince, apricots, peaches, plums, sweet and sour cherry. ** Commercial white tea; “silver needle” white tea; commercial green tea; “sencha” green tea; “matcha” green tea; commercial black tea; “Kenya” black tea; commercial Pu-erh tea; “Pu-erh”. Presented values correspond to the lower and higher contents determined among all samples. ^#^ Result presented as the sum of (a + a’) or (b + b’) isomers. ^##^ Intra-day variability results. ND—not detected.
